# Supplementary material for: Asynchronous magnetic resonance elastography: Shear wave speed reconstruction using noise correlation of incoherent waves
Source: Magn Reson Med. 2022 Oct 27;89(3):990–1001. doi: 10.1002/mrm.29502 (PMC9792433; doi:10.1002/mrm.29502)
Supplement: Supplementary file 1 — DATA S1 MATLAB code used to generate the numerical simulations of Figure 1 [file MRM-89-990-s002.zip › k-Wave/helpfiles/example_ivp_loading_external_image.html]

Loading External Image Maps Example (k-Wave)


# Loading External Image Maps Example

This example demonstrates how to assign an external image to the initial pressure distribution for the simulation of an initial value problem within a two-dimensional homogeneous propagation medium. It builds on the Homogeneous Propagation Medium Example.

- Open the file in the MATLAB Editor
- Run the file in MATLAB

## Contents

- Loading the image map

## Loading the image map

The initial pressure distribution `source.p0` used by `kspaceFirstOrder2D` is simply a two-dimensional matrix filled with arbitrary numeric values. Consequently, any data may be used to define this distribution. Here an external image map is loaded using `loadImage`. This function converts an external image into a matrix, sums the colour channels (for colour images), and scales the pixel values from 0 to 1.

```
% load the initial pressure distribution from an image and scale the
% magnitude
p0_magnitude = 3;
p0 = p0_magnitude * loadImage('EXAMPLE_source_one.png');
```

The medium discretisation is again performed by calling `kWaveGrid` with the required grid dimensions, after which `resize` is used to scale the loaded image map to the required dimensions.

```
% create the computational grid
Nx = 128;           % number of grid points in the x (row) direction
Ny = 128;           % number of grid points in the y (column) direction
dx = 0.1e-3;        % grid point spacing in the x direction  [m]
dy = 0.1e-3;        % grid point spacing in the y direction  [m]
kgrid = kWaveGrid(Nx, dx, Ny, dy);

% resize the image to match the size of the computational grid and assign
% to the source input structure
source.p0 = resize(p0, [Nx, Ny]);
```

The simulation is invoked in the same way as in the previous examples. A plot of the initial pressure distribution and sensor mask, and a visualisation of the recorded pressure field are shown below.
